# Unity in defence: honeybee workers exhibit conserved molecular responses to diverse pathogens

**DOI:** 10.1186/s12864-017-3597-6

**Published:** 2017-03-02

**Authors:** Vincent Doublet, Yvonne Poeschl, Andreas Gogol-Döring, Cédric Alaux, Desiderato Annoscia, Christian Aurori, Seth M. Barribeau, Oscar C. Bedoya-Reina, Mark J. F. Brown, James C. Bull, Michelle L. Flenniken, David A. Galbraith, Elke Genersch, Sebastian Gisder, Ivo Grosse, Holly L. Holt, Dan Hultmark, H. Michael G. Lattorff, Yves Le Conte, Fabio Manfredini, Dino P. McMahon, Robin F. A. Moritz, Francesco Nazzi, Elina L. Niño, Katja Nowick, Ronald P. van Rij, Robert J. Paxton, Christina M. Grozinger

**Affiliations:** 1 German Centre for Integrative Biodiversity Research (iDiv) Halle-Jena-Leipzig, Leipzig, Germany; 2 Centre for Ecology and Conservation, University of Exeter, Penryn, UK; 3 Institute of Computer Science, Martin Luther University Halle-Wittenberg, Halle (Saale), Germany; 4 Technische Hochschule Mittelhessen, Gießen, Germany; 5 INRA, UR 406 Abeilles et Environnement, Avignon, France; 6 Dipartimento di Scienze AgroAlimentari, Ambientali e Animali, Università degli Studi di Udine, Udine, Italy; 7 Institute of Life Sciences, University of Agricultural Sciences and Veterinary Medicine, Cluj-Napoca, Romania; 8 Department of Biology, East Carolina University, Greenville, NC USA; 9 Center for Comparative Genomics and Bioinformatics, Pennsylvania State University, State College, PA USA; 10 School of Biological Sciences, Royal Holloway University of London, Egham, Surrey UK; 11 Department of Biosciences, Swansea University, Swansea, UK; 12 Department of Plant Sciences and Plant Pathology, Montana State University, Bozeman, MT USA; 13 Department of Entomology, Center for Pollinator Research, Pennsylvania State University, State College, PA USA; 14 Department of Molecular Microbiology and Bee Diseases, Institute for Bee Research, Hohen Neuendorf, Germany; 15 Department of Microbiology and Epizootics, Freie Universität Berlin, Berlin, Germany; 16 Department of Fisheries, Wildlife, and Conservation Biology, The Monarch Joint Venture, University of Minnesota, St. Paul, MN USA; 17 Department of Molecular Biology, Umeå University, Umeå, Sweden; 18 Institute for Biology, Martin Luther University Halle-Wittenberg, Halle (Saale), Germany; 19 School of Biological Sciences, Queen’s University Belfast, Belfast, UK; 20 Institute of Biology, Freie Universität Berlin, Berlin, Germany; 21 Department for Materials and Environment, BAM Federal Institute for Materials Research and Testing, Berlin, Germany; 22 Department of Entomology and Nematology, University of California, Davis, CA USA; 23 Department of Computer Science, TFome Research Group, Bioinformatics Group, Interdisciplinary Center of Bioinformatics, University of Leipzig, Leipzig, Germany; 24 Paul-Flechsig-Institute for Brain Research, University of Leipzig, Leipzig, Germany; 25 Department of Medical Microbiology, Radboud Institute for Molecular Life Sciences, Radboud University Medical Center, Nijmegen, The Netherlands; 26 Present address: MRC IGMM, University of Edinburgh, Western General Hospital, Edinburgh, UK; 27 Present address: MRC Functional Genomics Unit, Department of Physiology, Anatomy and Genetics, University of Oxford, South Parks Road, Oxford, UK; 28 Present address: International Centre of Insect Physiology and Ecology (icipe), Environmental Health Theme, Nairobi, Kenya

**Keywords:** *Apis mellifera*, *Nosema*, *Varroa destructor*, DWV, IAPV, RNA virus, Meta-analysis, Transcriptomics, Co-expression network, Immunity

## Abstract

**Background:**

Organisms typically face infection by diverse pathogens, and hosts are thought to have developed specific responses to each type of pathogen they encounter. The advent of transcriptomics now makes it possible to test this hypothesis and compare host gene expression responses to multiple pathogens at a genome-wide scale. Here, we performed a meta-analysis of multiple published and new transcriptomes using a newly developed bioinformatics approach that filters genes based on their expression profile across datasets. Thereby, we identified common and unique molecular responses of a model host species, the honey bee (*Apis mellifera*), to its major pathogens and parasites: the Microsporidia *Nosema apis* and *Nosema ceranae*, RNA viruses, and the ectoparasitic mite *Varroa destructor*, which transmits viruses.

**Results:**

We identified a common suite of genes and conserved molecular pathways that respond to all investigated pathogens, a result that suggests a commonality in response mechanisms to diverse pathogens. We found that genes differentially expressed after infection exhibit a higher evolutionary rate than non-differentially expressed genes. Using our new bioinformatics approach, we unveiled additional pathogen-specific responses of honey bees; we found that apoptosis appeared to be an important response following microsporidian infection, while genes from the immune signalling pathways, Toll and Imd, were differentially expressed after *Varroa*/virus infection. Finally, we applied our bioinformatics approach and generated a gene co-expression network to identify highly connected (hub) genes that may represent important mediators and regulators of anti-pathogen responses.

**Conclusions:**

Our meta-analysis generated a comprehensive overview of the host metabolic and other biological processes that mediate interactions between insects and their pathogens. We identified key host genes and pathways that respond to phylogenetically diverse pathogens, representing an important source for future functional studies as well as offering new routes to identify or generate pathogen resilient honey bee stocks. The statistical and bioinformatics approaches that were developed for this study are broadly applicable to synthesize information across transcriptomic datasets. These approaches will likely have utility in addressing a variety of biological questions.

**Electronic supplementary material:**

The online version of this article (doi:10.1186/s12864-017-3597-6) contains supplementary material, which is available to authorized users.

## Background

Eukaryotes are natural hosts of multiple pathogens. Consequently, host immune systems have evolved efficient responses to threats of a different nature, such as viruses, bacteria or eukaryotic parasites. In vertebrates, adaptive immune mechanisms and antibody-mediated defences confer pathogen-specific responses [1]. Conversely, invertebrates lack these adaptive immune defences and rely primarily on innate immunity; they therefore have long been considered rather non-specific in their immune response. However, as insect immunological research has progressed, the specificity of insect antimicrobial action has become well established [2], with evidence of immune memory [3, 4] and pathogen-genotype to host-genotype interactions demonstrated in insect models [5].

Eusocial insects, including honey bees (*Apis mellifera*), establish large colonies comprised of thousands of related individuals, living at high density, sharing food in extended interactions and very high nest homeostasis; this lifestyle provides advantages in terms of social immunity [6] but also facilitates microbe transmission within the colony and promotes multiple infections [7]. Additionally, comparative genomics has revealed a loss of canonical immune genes in bees of social and solitary lifestyle compared to other insects such as flies, *Nasonia vitripennis* and *Tribolium castaneum*, that questioned the ability of bees’ immune system to respond specifically and efficiently to diverse, emerging pathogens [8]. Indeed, parasites and pathogens are considered one of the major factors driving global losses of honey bee colonies [9–11], which in turn threaten plant pollination, which is an important ecosystem service carried out by both managed and wild bees [12]. Key eukaryotic honey bee pathogens include two microsporidian gut parasites: *Nosema apis*, which primarily infects the Western honey bee *A. mellifera*, and *Nosema ceranae*, which was first described as a pathogen of the Eastern honey bee *Apis cerana* and more recently has become the predominant microsporidial pathogen infecting *A. mellifera* [13]. Single-stranded RNA viruses represent another key group of honey bee pathogens [14]. Several of these viruses are transmitted by *Varroa destructor*, an invasive ectoparasitic mite that switched host from *A. cerana* to *A. mellifera* in the past half century [15]. The ensuing shift of viruses from oral to vectored transmission by *Varroa* has modified the epidemiology and potentially increased the virulence of viral diseases such as deformed wing virus, thereby producing a significant threat to honey bee populations [16–18]. Importantly, multiple pathogens and parasites may interact while co-infecting honey bees to modify the dynamic of their infection [19, 20], and potentially increasing host mortality [17, 21].

Understanding the molecular interactions between the honey bee and its pathogens is crucial in revealing their role in host health and, ultimately, colony losses [22]. Recent advances in genome sequencing technologies and improvements in genome annotation of the honey bee have facilitated fine scale mapping of bee immune responses against multiple pathogens and parasites at the genomic level [23]. Several studies examining the transcriptional response of honey bees to their primary pathogens, namely *Nosema*, *Varroa* and viruses, have already provided considerable insight into the molecular mechanisms mediating host-parasite interactions [24–29], yet these studies have also revealed idiosyncrasies across datasets.

Analysis of multiple transcriptome datasets provides not only the opportunity to detect subtle changes in gene expression, but also to highlight commonalities in host responses. Recent studies in mosquitoes and humans have demonstrated the power of meta-analyses to reveal key host responses to multiple pathogen infections [30–32]. To comprehensively characterize the interactions between honey bees and their major pathogens and pests, we performed a meta-analysis of the transcriptional responses to *Nosema*, *Varroa* and viruses. We collected the gene expression profiles of 7,077 genes across 19 published and new transcriptome datasets of experimentally infected or parasitized honey bees, and utilized statistical and bioinformatics analyses that we newly developed (a ‘directed rank product analysis’) to perform a synthesis of gene expression patterns from multiple studies and platforms. This resulted in a robust analysis that, (i) identified common genes and pathways regulated in response to different pathogens, (ii) identified genes and pathways uniquely regulated in response to one pathogen in a particular body part or tissue, and (iii) enabled building a gene co-expression network to identify regulatory genes and new gene interactions within the honey bee transcriptome. Our analysis provides new insights into the molecular and physiological mechanisms that underpin the interactions between honey bees and their major pathogens.

## Results

### Multivariate analysis

We performed a multidimensional scaling analysis to visualize the spread of the 19 transcriptome datasets. This showed that gene expression levels vary less within a study than between studies and suggests that gene expression profiles are markedly influenced by experimental design (Additional file 1: Figure S1). Thus, comparisons across studies to find commonly and consistently regulated gene expression patterns are undoubtedly hindered by this large amount of variation, highlighting the importance of performing a meta-analysis with appropriate bioinformatics approaches to obtain robust and reproducible results.

### Rank product analysis

Previous comparative analyses of honey bee immune responses across transcriptome datasets simply involved determining if there was a significant overlap in the differentially expressed gene lists from different studies [24, 25, 27, 28]. However, the significant variation in expression levels between studies (Additional file 1: Figure S1) undoubtedly reduces the power of such comparisons. Thus, we employed a rank product analysis to identify sets of genes that are significantly differentially expressed across the 19 transcriptomes datasets we collected. The rank product analysis is a non-parametric statistic that identifies genes that are consistently highly ranked in a number of datasets, based on the gene expression fold changes.

**Fig. 1:**
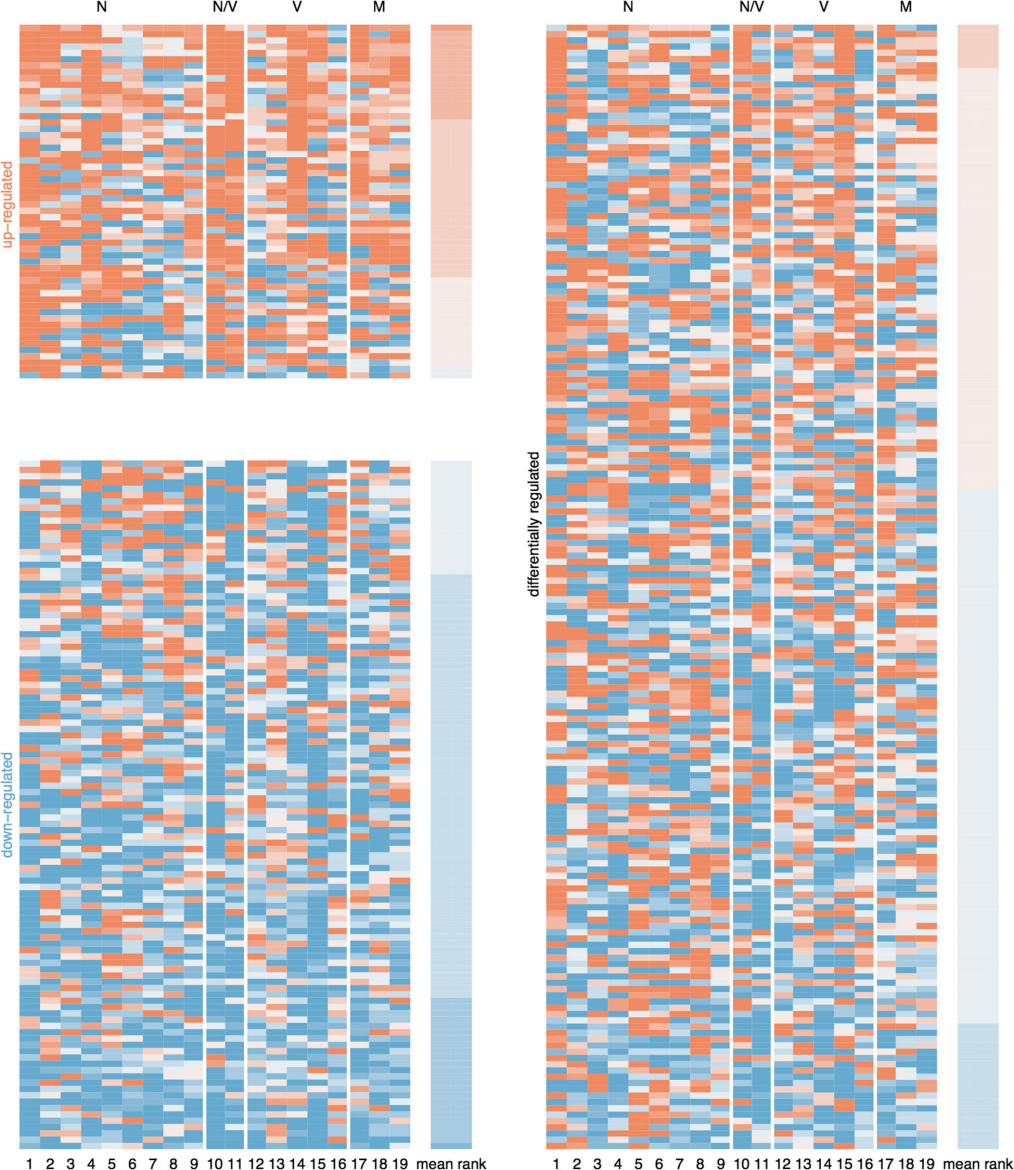
Heat maps illustrating the expression levels (relative ranks) of the 344 significantly regulated genes across the 19 transcriptome datasets. Genes are categorized as 56 up-regulated genes (*top left*), 109 down-regulated genes (*bottom left*), and 179 differentially regulated (*up* and *down*) genes (*right*). *Orange* shows increased expression and blue decreased expression after pathogen infection. Top classification is N for *Nosema* infection, N/V for *Nosema* and RNA virus co-infection, V for virus, and M for *Varroa* mite (‘*Varroa* plus virus’). Numbers at the bottom correspond to dataset numbers in Table 2. Each row represents the differential expression of the same gene across all 19 datasets. In each category, genes are ordered following the arithmetic means of their ranks displayed in the right column of the heat map. Note the presence of genes showing decreased expression in some datasets although found as statistically up-regulated across datasets, and vice-versa

In total, we found 344 genes with significant differential expression across datasets, categorized by (i) 56 genes with significant increased expression (i.e. up-regulated) across datasets, (ii) 109 with significant decreased expression (i.e. down-regulated) across datasets and (iii) 179 genes with significant differential expression (i.e. differentially-regulated), up-regulated in some studies, down in others (Fig. 1; Additional file 1: Figures S2 and S3; Additional file 2: Tables ST1-ST3). Note that using this rank product analysis, a gene may be statistically significantly up-regulated across all 19 datasets but still be down-regulated in one or more datasets (and vice-versa for significantly down-regulated genes). In fact, subsets of up-regulated genes (45 of 56 genes) and down-regulated genes (83 of 109 genes) were also categorized as differentially-regulated (up- and down-regulated across datasets; see Additional file 1: Figure S3). Notably, one gene, encoding the antimicrobial peptide (AMP) *hymenoptaecin*, was present in all three categories due to its extreme differential expression (high and low) across all datasets (Additional file 1: Figure S4).

### Gene evolutionary rate

**Fig. 2:**
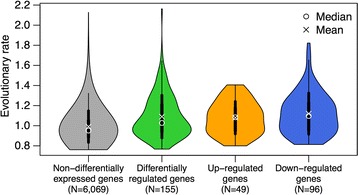
Comparison of the evolutionary rate between genes showing significant differential expression and genes without significant differential expression across the 19 datasets. Relative evolutionary rates on the Y-axis are quantified from pairwise alignments of the protein sequences, and represent the average of inter-species protein sequence identities normalized to the average identity of all inter-species orthologs from OrthoDB [33]. The *vertical black lines* along the median and mean values of each category represent the standard deviation (*thick lines*) and the 95% confidence intervals (*thin lines*). Horizontally, the width of each violin box represents the density of the data values, i.e. the distribution of the data along the y axes, for each category

We compared the evolutionary rate, obtained from the database OrthoDB and measured as the average of protein sequence identities from pairwise alignments across 12 bee genomes [33], of genes that were significantly differentially expressed across the transcriptome datasets and genes that were not differentially expressed. Genes showing significant differential expression across the transcriptome datasets exhibited a higher evolutionary rate than non-differentially expressed genes (Kruskal-Wallis *χ* ^2^ = 103.1476; df = 3; *p* < 0.001; Fig. 2), suggesting rapid evolution of genes responding to pathogen infection. All three categories of differentially expressed genes showed significantly higher evolutionary rates than non-differentially expressed genes (Dunn’s test with Benjamini-Hochberg corrected *p*-values: differentially-regulated vs. non-differentially expressed Z = −6.536, *p* < 0.001; up-regulated vs. non-differentially expressed Z = −4.183, *p* < 0.001; down-regulated vs. non-differentially expressed Z = −6.781, *p* < 0.001), while no difference was observed between the different categories of regulated genes. Note that an analysis of the dN/dS ratios may have provided more insights into the type of selection (e.g. positive, relaxed) but this was beyond the scope of this manuscript.

### Specific host responses

We explored the specific response of honey bees to pathogens using our newly developed directed rank product analysis. By this method, we adjusted the rank product analysis approach to identifying genes whose expression followed a specific pattern. This new method consists in defining a theoretical gene expression profile corresponding to selected parameters (e.g. treatment, development time or tissue-specific responses), and identifies genes exhibiting a similar expression profile. Here, we identified genes differentially expressed in bees infected by one pathogen type but non-differentially expressed in bees infected by other pathogens.

We first identified genes specifically involved in the response to *Nosema* infection in abdominal tissues: midgut, fat body, or complete abdomen (Additional file 2: Tables ST4 and ST5). The functional analysis of 104 genes with increased expression upon *Nosema* infection revealed overrepresentation of genes encoding enzymes and proteins involved in metabolic processes, catalytic activities, and transporter activity (Additional file 2: Table ST6), while genes related to cell components were overrepresented among 88 down-regulated genes after *Nosema* infection (Additional file 2: Table ST7).

To explore the specific response of honey bees to viruses, we examined the transcriptome datasets of honey bees experimentally infected by RNA viruses or parasitized by *Varroa* mites and, thus, by viruses. We justify merging *Varroa* and virus datasets with the idea that the impact of *Varroa* may stem largely from damage to the cuticle during feeding as well as from transmitted viruses, thus suggesting little immediate impact of sole *Varroa* parasitism on immune gene expression of the host [34]. We identified 167 genes differentially expressed specifically in response to *Varroa*/virus treatments (88 up-regulated and 79 down-regulated; Additional file 2: Tables ST8 and ST9). The functional analysis of genes regulated after parasitism by *Varroa* and infection by viruses did not show any significantly overrepresented GO terms for up-regulated genes, but the overrepresentation of nutrient reservoir activity for down-regulated genes (Additional file 2: Tables ST10 and ST11).

### Gene co-expression network

**Fig. 3:**
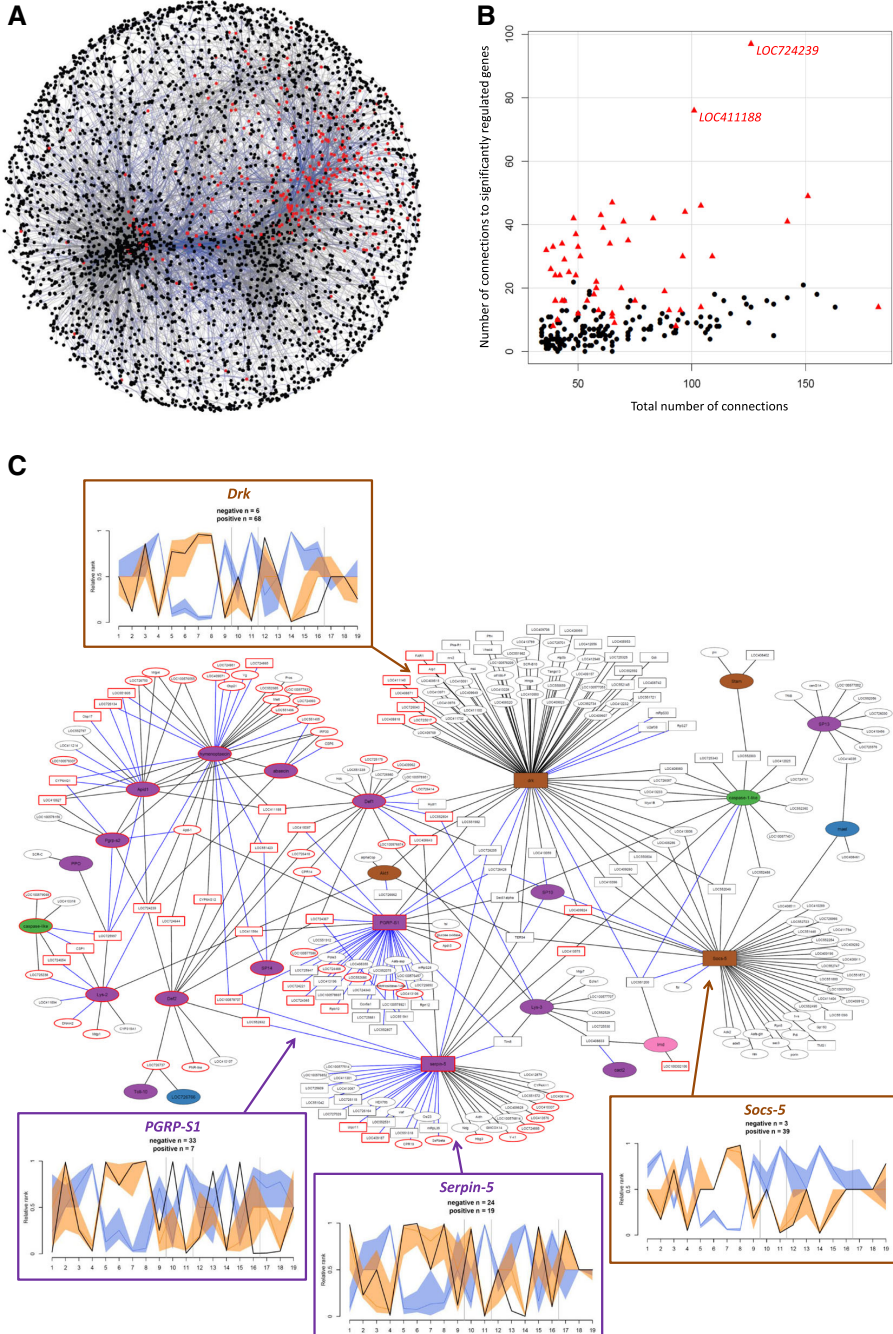
Gene co-expression network. **a** Main module of the gene co-expression network, representing 3,589 interconnected genes. Red nodes show genes significantly regulated across the 19 transcriptome datasets, and black nodes show non-significantly regulated genes. Square nodes show the most connected (hub) genes. Grey edges illustrate positive correlation between two gene expression profiles while blue edges show negative correlations. A file available at https://idata.idiv.de/DDM/Data/ShowData/35 provides the possibility of navigating within the network. **b** Scatter plot representing the total number of connections (x-axis) over the number of connections to significantly regulated genes across the 19 transcriptome datasets for the most (top 5%, *N* = 209) connected genes (i.e. hub genes). Red triangles show significantly regulated hub genes, while black dots show non-significantly regulated hub genes. Two hub genes with high connectivity to significantly regulated genes are shown: a kynurenine/alpha-aminoadipate aminotransferase (*LOC724239*), and a L-lactate dehydrogenase (*LOC411188*). **c** Main module from the co-expression network of the immune genes of the honey bee. Coloured nodes represent immune genes from the Toll (purple), JAK/STAT (brown), apoptosis (green), RNAi (blue) and Imd (pink) pathways (see immune genes list in the Additional file 2: Table ST13). Oval nodes show genes with low connectivity, squares show genes with high connectivity (hub genes, with at least 34 connections). Genes significantly regulated across the 19 transcriptome datasets have a red outline. Black edges represent positive co-expression and blue edges are negative co-expression. In insets, the expression profiles across the 19 transcriptome datasets (*black lines*) of the four immune hub genes (i.e. highly connected immune genes), accompanied by expression profiles of genes with which they are connected. Orange profiles display similar profiles (positive connections, i.e. *black lines* in the network) and blue reflect opposite profiles (negative connections, i.e. *blue lines* in the network). The y-axis displays the relative ranks of differential expression level, from up-regulated (value towards 1) to down-regulated (value towards 0)

Our directed rank product method enabled us to detect genes with similar and opposite expression profiles across the 19 transcriptome datasets and to build a gene co-expression network. We found a total of 16,110 significant inter-gene connections, with nearly half of the 7,077 genes (*N* = 3,589) interconnected within one major module, while 2,931 genes remained unconnected and 557 genes were placed within small modules of 2 to 11 genes. The majority (98%) of inter-gene connections were observed within the major module, with 12,694 positive (i.e. similar expression profiles) and 3,087 negative (i.e. opposite expression profiles) inter-gene connections (Fig. 3a). Notably, 320 out of the 344 significantly differentially expressed genes from the rank product analysis were identified within the major module, illustrating the tight interconnectivity of the expression regulation of these genes.

We identified the top 5% most interconnected genes from all 7,077 genes of this study, which represents 209 hub genes with at least 34 connections to other genes (Additional file 1: Figure S5). Notably, 52 hub genes were significantly differentially expressed across the 19 transcriptome datasets (Fig. 3b; Additional file 2: Table ST12), and differentially expressed genes were significantly more connected than non-differentially expressed genes (Kruskal-Wallis *χ* ^2^ = 445.9856, df = 3, *p*-value < 0.001; Additional file 1: Figure S6).

To identify novel candidate genes involved in immune regulation, we collected all interactions involving canonical immune genes and generated an immune network composed of 26 modules containing at least 2 interconnected genes. The major module of this immune network contained 281 genes, including 25 immune genes from all immune pathways (Fig. 3c; Additional file 2: Table ST13). A total of 92 significantly regulated genes from the rank product analysis were present in this immune network, with only one remaining outside the major module.

## Discussion

**Fig. 4:**
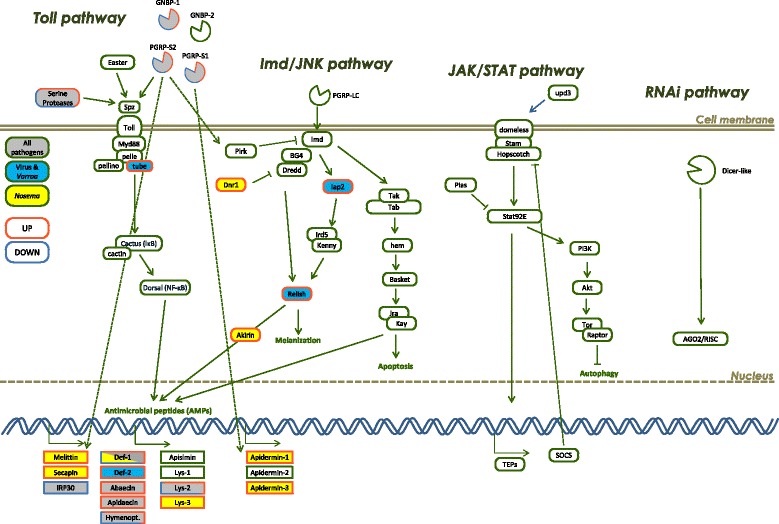
Diagram of the canonical innate immune response of the honey bee. Gene names in colour-filled boxes show evidence of significant regulation after infection by *Nosema* (yellow), or infection by RNA viruses and/or infestation by *Varroa* mites (light blue) or all pathogens (grey). *Orange lines* surrounding a box show increased expression and blue surrounding lines indicate decreased expression after pathogen infection –mixed orange and blue lines show genes found differentially-regulated, either up- or down-regulated across the datasets. Notably, the AMP *defensin-1* exhibited increased expression in most of the datasets, but a decreased expression in the abdominal tissues of honey bees infected by *Nosema*. Therefore, a mixed background and outline colour are displayed. *Green* surrounding lines show genes found non-significantly regulated in this analysis. *Solid lines* with arrows show gene interactions reported in the literature, and *dotted arrows* indicates new potential interactions inferred from our gene co-expression network analysis

Similar to other eukaryotes, the honey bee is host to many different types of pathogens and harbours in its genome an immune repertoire to provide a specific immune response to this diversity of pathogens [35]. Our meta-analysis of honey bee transcriptional responses to a diverse set of pathogens identified a core set of genes that is common to honey bee anti-pathogen responses, as well as suites of genes that respond specifically to different pathogens and parasites (Fig. 4).

### General expression patterns

Multivariate analysis indicated that there was a wide between-study variation, likely due to a combination of factors, including variation in technology, experimental approach (e.g., RNA extraction method, infection dose), tissues examined, method of analysis, and host and pathogen genotypes. These substantial differences may explain the limited overlap observed previously between differentially expressed gene lists from different studies [24, 25, 27, 28]. This underscores the importance of our approach, which aims to provide a general methodological and statistical synthesis of studies in order to reveal commonalities in host response to pathogen invasion. It is also a cautionary note for the interpretation of gene expression patterns from single experimental studies, which may in part be due to the idiosyncrasies of a specific experimental paradigm.

### Common host response

The common response to multiple pathogens identified in our meta-analysis is characterized by the increased expression of several immune genes, including all canonical AMPs, genes encoding cuticular proteins (*LOC552685*, *CPR14* and *LOC100577229*), which likely respond to tissue damage by pathogens, and heat-shock related proteins (*LOC410087*, *LOC724367*), which may serve as markers of stress during infection response [36]. However, substantially more genes showed decreased expression across transcriptome datasets in response to pathogen infections. Among them we found genes encoding enzymes involved in carbohydrate metabolism, four cytochrome oxidase P450s, the egg yolk protein precursor vitellogenin (*Vg*), the major royal jelly proteins *Mrjp1* and *Mrjp9* and two transcriptional repressors *hairy* and *knirps*. This reduced expression of genes involved in catalytic and metabolic activities may illustrate the cost of the infection, i.e. a dysregulation as a consequence of pathogen insult as opposed to host adaptive response, or a manipulative response of the host by the pathogen to enhance its own replication. However, several genes regularly reported as responding to pathogen assault were also down-regulated; these genes encode serine proteases, GMC oxidoreductases, *Toll-like receptor 13*, the putative antimicrobial peptide *IRP30*, and *glucose oxidase*, an enzyme involved in colony food sterilisation and a major component of social immunity in honey bees [37]. Down-regulation of immune genes might represent an adaptive manipulation of the host by infecting pathogens, although this remains to be demonstrated.

An additional 179 genes showed significant differential expression but were inconsistently up- and down-regulated across the 19 transcriptome datasets. Among them we found many cytochrome oxidase P450s and immune genes, including the recognition receptors *PGRP-S1, PGRP-S2*, *B-gluc1*, *SP12* and *Serpin-5*, and the antimicrobial *Lys-2*, the gene encoding *caspase-like*, involved in apoptosis, and *ninjurin-1-like*, a transmembrane protein induced by the Toll immune pathway and involved in non-apoptotic cell death in *Drosophila* [38]. Other genes with potential immune activity also showed significantly variable regulation, such as the *GMC oxidoreductases 3* and *7*, the apidermins *Apd-2* and *Apd-3* and the hemolymph *apolipophorin-III-like protein*.

Functional analysis of the 344 significantly regulated genes showed an overrepresentation of genes associated with extracellular regions and response to biotic stimulus (e.g. immune genes involved in response to pathogen invasion), metabolic processes, and nucleotide binding activity (Table 1). Strikingly, breaking down the functional analysis into genes with increased expression and genes with decreased expression, we found no significant GO terms linked to genes with increased expression, while immune, metabolic and regulatory functions were significantly overrepresented in down-regulated genes. Whether this is an adaptive response of the host or a face of immune suppression driven by pathogens is currently not possible to determine.

**Table 1:** Functional analysis of significantly regulated genes across transcriptome datasets

Groups	GO terms	*p*-value
All significantly regulated genes	extracellular region	1.3E-06
	metabolic process	3.2E-04
	electron carrier activity	3.6E-04
	cellular protein modification process	3.6E-04
	response to biotic stimulus	3.2E-03
	protein complex	6.5E-03
	nucleus	1.4E-02
	carbohydrate metabolic process	1.4E-02
	nucleobase-containing compound metabolic process	1.4E-02
	catalytic activity	1.4E-02
	nucleotide binding	1.4E-02
	nucleic acid binding	1.6E-02
	regulation of biological process	1.6E-02
	protein kinase activity	2.3E-02
	transporter activity	2.9E-02
	signal transduction	3.0E-02
	cell cycle	3.0E-02
Up-regulated	*no significant terms*	
Down-regulated	extracellular region	1.1E-05
	regulation of biological process	6.4E-03
	response to biotic stimulus	6.4E-03
	electron carrier activity	1.0E-02
	metabolic process	1.9E-02
	nucleotide binding	4.9E-02
Differentially-regulated	metabolic process	1.5E-02
	catalytic activity	1.5E-02
	extracellular region	1.5E-02
	electron carrier activity	1.5E-02
	cellular protein modification process	3.7E-02

This table shows the overrepresented GOslim terms for all regulated genes (344 genes) and the categories; up-regulated, down-regulated and differentially-regulated. Note that no overrepresented GO term was obtained for up-regulated genes. Gene lists corresponding to these GO terms are available in Additional file 2: Tables ST1-ST3

Interestingly, the common use of immune genes against different types of pathogens may be a consequence of the reduced set of canonical immune genes observed in bees relative to other insect taxa [8], and may reflect a need for a more general rather than specific response against multiple pathogens. However, common host responses have also been described in other models, such as in mosquitoes and humans [30–32]. In humans, this general response to pathogens has been suggested to constitute an ‘alarm signal’, which may be triggered by different cell types, to maximize the detection and the response of infection [31]. The role of the common host response in honey bees remains to be determined.

Genes involved in this common host response (including several immune genes such as *hymenoptaecin*, *def-2*, *PGRP-S1*, *B-gluc1*) showed higher evolutionary rate among bees than genes which did not show significant expression differences across the transcriptome datasets. Notably, *B-gluc1* has recently been shown to be evolving under positive selection in the honey bee [8]. Recent analysis suggested that sociality and the increased pathogen pressure in colonies densely packed with worker bees may be a cause of such rapid evolution in bumble bee immune genes [39]. However, rapid evolution of immune genes may not only arise as a consequence of positive evolution, but also from relaxed selection [40]. Indeed, honey bees do not rely exclusively on canonical immune genes to fight pathogen infections, but can also employ other mechanisms, such as social immunity [6].

### Specific response to pathogens

To characterize the specific response of the honey bee to its major pathogens, we identified genes whose expression profile across transcriptome datasets is most similar to a theoretical expression profile, i.e. genes that are up or down-regulated in response to a pathogen type, Microsporidia or *Varroa*/virus. This method, named here ‘directed rank product analysis’, combines the identification of genes following a specific expression pattern by subtraction of a gene’s differential expression scores (i.e. here, relative ranks), within the statistical framework of the rank product analysis. This method takes advantage of the rank product analysis so that it can detect biologically relevant gene expression changes from heterogeneous datasets obtained from different platforms, microarrays and sequencing [41].

Despite the common gene expression response identified above, we found important differences in the transcription responses of honey bees to *Nosema* and *Varroa*/virus infections. The specific response to *Nosema* includes increased expression of several genes involved in the regulation of cell death by autophagy or apoptosis, such as *Atg2*, *LOC409667*, *Metap2* and the apoptosis inhibitor *dnr1*, which confirms the importance of these mechanisms in mediating the interaction between the honey bee and Microsporidia [42, 43]. Other immune genes were up-regulated upon *Nosema* infection, including: the transcriptional co-factor *akirin* and *lys-3*, involved in the Imd pathway, *laccase-2*, important for melanisation [44] and the venom proteins *melittin* and *secapin*, known for their antimicrobial activities [45, 46]. Conversely, the expression of AMP *Def-1* and the serine protease *SP40* were reduced in *Nosema* infected honey bees. Two chitin-binding genes showed contrasting response to *Nosema* infection: while chitinase 5 (*Cht5*) exhibited increased expression, the cuticular protein *chitotriosidase-1* exhibited reduced expression. This may reflect either a direct effect against *Nosema* or a response to tissue damage induced by the pathogen.

The specific transcriptional-level response against *Varroa*/virus treatments was characterized by the differential expression of genes from the Imd (*iap2* and *rel*) and Toll pathways (*tube* and *def-2*). Although historically described as anti-bacterial and anti-fungal [2], these pathways were recently shown to exhibit differential expression upon viral infection, and potentially playing active roles in the antiviral defence of insects [47], including in honey bees [17, 28, 29, 48, 49]. Particularly, the Toll pathway NF-κB homolog *dorsal-1A* was shown to be transcriptionally induced in worker honey bees parasitized by *Varroa* mites, suggesting that *dorsal-1A* is involved in the control of DWV infections [17]. While activation of the Imd and Toll pathways induces higher expression of AMPs, the antiviral roles of AMPs are not well characterized [47]. Alternatively, these pathways may possibly control the proliferation of haemocytes, which are important for phagocytosis in the insect cellular immune response, and potentially play a role in the antiviral response [50]. Changes in expression levels of AMPs after virus infection may be a consequence of activation of the Toll and Imd pathways, without having a direct functional role against viruses.

Importantly, we did not observe increased expression of genes associated with the RNAi pathway (e.g. *Dicer*, *Ago*), though this is an important component of the antiviral response in insects [51, 52] and observed in two studies included in this meta-analysis [24, 28]. The action of the RNAi pathway may be transient, and thus not always captured by transcriptome analysis. Up-regulation of these genes may be detectable only during the early stages of viral infection, which would explain an inconsistent effect in our dataset, since host transcriptomes were measured at different times post-infection. We also found the increased expression of genes encoding a transcription factor (*LOC727085*), translation factors (*EF1-alpha*, *LOC726500*) and post-transcriptional modification proteins (*LOC412975*, *LOC724690*), which may illustrate a general transcriptome dysregulation following infection by viruses [53]. Finally, we found the gene encoding *Vg* to be down-regulated following viral infection, which may reflect a lack of regulation of nutrients and/or an impaired physiology of the host [54]. *Vg* is also known to mediate the immune response in honey bees [55]. Importantly, lower expression of *Vg* and increased expression of *malvolio* (*Mvl*) –also observed in response to *Varroa*/virus– are associated with accelerated behavioural maturation and foraging activity in worker honey bees [56, 57]. The altered expression levels of these regulators in infected individuals, and subsequent induction of precocious foraging, is likely an adaptive response against pathogen transmission within the colony [58], one of many potentially adaptive behavioural responses against pathogens [6].

### Gene co-expression network

In addition to identifying shared and unique responses to pathogens, our large dataset enabled the exploration of gene co-expression and the identification of new regulatory genes. Among the most interconnected (hub) genes, we found several genes encoding ribosomal proteins and NADH dehydrogenase enzymes. But most importantly, we identified two genes with many inter-gene connections with other differentially expressed genes: a kynurenine/alpha-aminoadipate aminotransferase gene (*LOC724239*) and an L-lactate dehydrogenase gene (*LOC411188*), exhibiting 97 and 76 connections, respectively. The expression of both genes were significantly increased in most transcriptome datasets, and connected to a large proportion of genes that exhibited reduced expression (65 and 50% of negative interactions, respectively). We hypothesize that they exert considerable influence on the overall transcriptional response to pathogen infection and thus may be important mediators of the common host response against diverse pathogens. Interestingly, the aminotransferase *LOC724239* was recently shown to be involved in trans-generational immune priming in the bumblebee *B. terrestris* [59], also suggesting a putative immune regulation function in this species.

More specifically, our immune gene co-expression network highlights the interconnection of all immune pathways. We observed the tight co-expression of the genes encoding canonical AMPs, together with other genes with antimicrobial properties (*Lys-3*, *melittin*, *IRP30*), suggesting a concomitant action after pathogen invasion and/or an identical regulatory mechanism. Expression of these AMPs was positively correlated with expression of the genes encoding the recognition protein *PGRP-S2* and the serine protease *SP14*, both of which are involved in signalling within the Toll pathway [35]. Importantly, new immune genes were identified, including the ortholog of the *Drosophila* gene *pirk* (*LOC100578156*), a negative regulator of the immune Imd pathway [60], co-expressed here with *PGRP-S2*.

Four immune genes are amongst the most connected genes: *PGRP-S1*, *Serpin-5*, *Socs-5* and *Drk. PGRP-S1* and the serine protease inhibitor *Serpin-5*, involved in the Toll pathway, are known for their immune regulatory activities [35]. In the immune network generated by our meta-analysis, this activity is illustrated by their numerous negative correlations with the expression profiles of other genes. The suppressor of cytokine signalling, *Socs-5*, is also known to have a negative feedback effect on the JAK/STAT pathway [35]. However any putative immune regulation by *Drk*, which is important for the activation of MAPK signalling in *Drosophila* [61], remains unclear. The mechanisms and possible applications behind these large regulatory effects within the honey bee immune system remain to be investigated.

## Conclusions

The accumulation of genome-wide studies has provided the opportunity for the analysis of the commonalities and idiosyncrasies in gene expression in host response to pathogen attack. Here, we synthesised 19 transcriptome datasets from experimentally infected honey bees and developed a new bioinformatics method, the directed rank product, to analyse gene expression profiles in order to identify the host specific responses to a diverse set of pathogens, and build a robust co-expression network. Although this method does not account for the amplitude of gene expression changes, using a rank product-based analysis has the advantage of enabling use of data from different platforms (e.g. microarray and RNA sequencing) in a single statistical analysis.

Importantly, our analyses revealed a core set of genes involved in a common host response to phylogenetically distinct pathogens, yet also enabled identification of genes involved in pathogen specific host immune responses. For instance, we showed that conserved pathways are involved in response to multiple pathogens, with the cellular immune response playing a key role in interactions with *Nosema* in abdominal tissues, while humoral immune pathways seem to have important antiviral activities. This analysis also broadens the definition of honey bee immune response by identifying genes encoding proteins, such as *melittin* and *secapin*, which have not been considered part of the canonical immune response. Using a gene co-expression analysis, we also identified potentially important mediators and regulators of anti-pathogen responses, including the Toll-pathway genes *Serpin-5* and *PGRP-S1*, the JAK/STAT modulators *Drk* and *Socs5*, and the newly characterized kynurenine/alpha-aminoadipate aminotransferase (*LOC724239*) and an L-lactate dehydrogenase (*LOC411188*). Overall, our synthesis helps to pinpoint key host genes and pathways that respond to phylogenetically diverse pathogens. This gene list will likely be an important source for future functional studies and potentially for selecting more resilient honey bee stocks [23, 62]. More generally, the statistical and bioinformatics approaches developed in this study can be broadly applied to synthesize information across transcriptomic datasets to address a wide array of biological questions.

## Methods

### Dataset selection for meta-analysis

We restricted our analysis to microarrays and RNA-seq datasets obtained from experimentally infected honey bee workers (Table 2). In total, we collected 19 transcriptome datasets obtained from nine experiments, reporting the differential expression of transcripts between control bees and samples parasitized by *Nosema* spp., RNA virus and/or *V. destructor* and in which pathogen infection was a formal component of the experimental design (i.e. studies in which transcriptomes were generated for control and treatment groups). These 19 datasets were either from unpublished studies generated by the co-authors or recently published (and therefore publicly available) studies at the start of our work. Microarray probes and gene identifiers were converted or updated to the latest version of the honey bee genome assembly Amel_4.5 and its annotation from NCBI [63]. Differential gene expression data (treatment vs. control) were provided by authors of studies in terms of log_2_ fold changes.

**Table 2:** List of the 19 transcriptome datasets

#	Parasite	Cat.	Age (days)	Days p.i.	Tissue	Technology	Reference
1	*N. ceranae*	N	15	13	Brain	RNA-seq	[24]
2	*N. ceranae*	N	10	10	Brain	RNA-seq	[25]
3	*N. ceranae*	N	14	7	Midgut	Tiling array	[26]
4	*N. ceranae*	N	13	12	Abdomen	Microarray	[74]
5	*N. apis*	N	3	1	Midgut	Microarray	[27]
6	*N. apis*	N	8	2	Midgut	Microarray	[27]
7	*N. apis*	N	2	2	Fat body	Microarray	[27]
8	*N. apis*	N	3	7	Fat body	Microarray	[27]
9	*N. apis* and *N. ceranae* ^a^	N	15	14	Fat body	Microarray	[27]
10	*N. ceranae* and BQCV ^a^	N/V	15	13	Brain	RNA-seq	[24]
11	*N. ceranae* and DWV ^a^	N/V	13	12	Abdomen	Microarray	[74]
12	SINV-GFP ^b^	V	4	3	Whole bee	Microarray	[29]
13	DWV	V	pupae	3	Brain	Microarray	[75]
14	DWV	V	13	12	Abdomen	Microarray	[74]
15	BQCV	V	15	13	Brain	RNA-seq	[24]
16	IAPV	V	1	1	Fat body	RNA-seq	[28]
17	*V. destructor* ^*c*^	M	10	-	Brain	RNA-seq	[25]
18	*V. destructor* (*N* = 1 mite) ^d^	M	1	12	Whole bee	RNA-seq	[76]
19	*V. destructor* (*N* = 3 mites) ^e^	M	1	12	Whole bee	RNA-seq	[76]

All datasets were generated from worker honey bees experimentally infected by *Varroa* mites, RNA viruses and/or *Nosema* spp., and for which gene expression was compared with uninfected control samples. Note that *Varroa* parasitism was also associated with high viral titers and therefore represented a ‘*Varroa* plus virus’ treatment. *BQCV* black queen cell virus, *IAPV* Israeli acute paralysis virus, *DWV* deformed wing virus. Categories (Cat.) are N for *Nosema*, N/V for *Nosema* and virus co-infection, V for virus alone and M for *Varroa* mite (‘*Varroa* plus virus’), as used across this study. Age and Days p.i. gives the age (i.e. days post-eclosion) and the number of days post infection when bees were collected for transcriptome analysis

^a^ Studies where honey bees were co-infected with two pathogens

^b^ This study used the model Sindbis virus expressing enhanced green fluorescent protein (SINV-GFP)

^c^ Transcripts from DWV (4 to 15 × 10^5^ tags) and Varroa destructor virus (21 to 25 × 10^6^ tags) present in brain transcriptomes from *Varroa* infested bees

^d^ Average proportion of reads attributed to DWV = 37.6% (±14.8 sem)

^e^ Average proportion of reads attributed to DWV = 47.7% (±17.7 sem)

The use of one dataset (#3 in Table 2) required the reprocessing of the original raw data. We retrieved the pre-processed tiling array expression data (GSE25455) from NCBI GEO as described by Dussaubat et al. [26]. We then re-annotated the probe sequences of the tiling array by alignment to *Apis mellifera* transcripts extracted from Amel_4.5 annotation as in Poeschl et al. [64]. We used the re-annotated probes to create sets of probes to measure the abundance of each transcript. We extracted the already computed log fold changes from the data files and applied quantile normalization. We used the new probe annotation to compute the median log_2_ fold change of all probes assigned to represent a transcript. We recovered log_2_ fold changes for 10,002 transcripts from three biological replicates.

### Gene annotation

Genes were annotated with GO terms using Blast2GO [65]. The first step of sequence alignments was done in-house using BLAST [66]. The sequences of transcripts associated with gene identifiers from the honey bee genome assembly were recovered and compared to those in the non-redundant database [67] [downloaded on 2014/03/06, containing 35,149,712 sequences] using *Blastx* (parameters: e-value cutoff of 1E-6 and maximum number of alignments 20). Alignments were uploaded to the Blast2GO server and all following steps were done according to the Blast2GO pipeline using default settings. GoSlim-terms were chosen for annotation to reduce redundancy among overrepresented GO terms [68].

### Selection of genes for inclusion in the analysis

We combined log_2_ fold expression values of 11,165 genes from the 19 transcriptome datasets in one *full synthesised dataset (fsd)* file. Due to the diversity of expression detection platforms and changes in gene annotation within recent years, 56% of the genes contained missing values in at least one transcriptome dataset; hence, only 44% of the genes with complete observation across the 19 datasets would remain for the analysis. Therefore, to increase the number of genes to include in our synthesis, we constructed a *restricted synthesised dataset (rsd)* with a subset of the *fsd* containing log_2_ fold expression values of 7,077 genes that had no more than three missing values (NAs) across the 19 transcriptome datasets. This enabled us to evaluate the expression changes of 63% of the annotated genes from the current genome assembly (see Additional file 1: Figure S7) and constituted the dataset for further analyses.

Statistical analysis of gene expression required complete observations of a gene across all 19 transcriptome datasets. To overcome missing data for genes with incomplete observations, we ordered gene expression values by their log_2_ fold change values and gave each a relative rank in each of the 19 transcriptome datasets. Relative ranks ranged between 1 for up-regulated genes and 0 for down-regulated genes. Missing values (5,015 of 134,463 in total) were then replaced by the average of non-missing relative ranks for the same gene from other datasets. We refer to this relative ranked dataset as ranked *rsd*, which is publicly available with the *fsd* at https://idata.idiv.de/DDM/Data/ShowData/35.

### Multidimensional scaling analysis

We visualized the spread of the datasets by performing multidimensional scaling using the ranked *rsd* values from the differential expression values of the 7,077 genes across the 19 transcriptome datasets. We computed the Manhattan distances between each pair of transcriptome datasets using the *cmdscale* function of the *stats* R package [69].

### Rank product analysis

Gene expression measurements using either microarrays or whole transcriptome sequencing (RNA-seq) vary greatly in methodology [70], resulting in substantial differences in the data produced. RNA-seq is generally more sensitive, producing gene expression levels spanning a greater dynamic range of values and resulting in a broad range of differential gene expression levels between control and infected samples (Additional file 1: Figure S8). In contrast, microarrays typically report lower differential gene expression.

To overcome this issue and compare a gene’s level of expression across different datasets regardless of its differential expression range, we performed a rank product analysis, a non-parametric statistic used for detecting differentially expressed genes based on (log) fold changes. The rank product analysis identifies genes that are consistently highly ranked in a number of datasets, and is a powerful approach to detect biologically relevant gene expression changes from heterogeneous datasets [41]. For this we used the *RankProd* R package [71], which accepts pre-processed expression datasets produced from different platforms, and thus was appropriate for our ranked *rsd*. In short, all 7,077 genes were ordered based on their relative ranks and were given ranks from 1 to 7,077 in each transcriptome dataset. From these new rank values the rank product was computed for each gene across the 19 transcriptome datasets and p-values were assigned to genes using a permutation test (*N* = 10,000) to test for differential expression. We then corrected for multiple testing using the Benjamini-Hochberg procedure from the *multtest* R package [72]. Using this approach, we identified genes that were (i) significantly up-regulated (genes with corrected rank product p-value < 0.05), (ii) genes significantly down-regulated (corrected p-value < 0.05), and (iii) genes significantly differentially-regulated regardless of the orientation (up-regulated in some studies, down in the others, and here termed differentially-regulated; corrected p-value < 0.05), across the 19 transcriptome datasets. For (i) and (ii) the ranked *rsd* was used to perform the analyses; note that a gene may be statistically significantly up-regulated across all 19 datasets by the rank product analysis but still be down-regulated in one or more datasets (and vice-versa for significantly down-regulated genes). For (iii) we transformed the data as follows: the log_2_ fold changes of each dataset were shifted by their median to obtain an equal amount of up- and down-regulated genes, to avoid biased analysis towards strongly up or down-regulated genes. Signs of the log_2_ fold changes were omitted and genes from group (iii) were first processed as described for the *rsd*, and then used to perform the analysis. Group (iii) therefore also contained genes of groups (i) and (ii).

This resulted in three lists of genes and their ranks in the 19 transcriptome datasets, their rank product value, p-value as well as corrected p-value. To test whether a specific GO term was significantly over- and under-represented in a specific gene set compared to a background set, we performed a two-sided Fisher’s exact test using the *stats* R package [69].

### Gene evolutionary rates

We collected from the OrthoDB open source [33] the relative evolutionary rates, calculated from 12 bee genomes (*Apis cerana*, *A. dorsata*, *A. florea*, *A. mellifera*, *Bombus impatiens*, *B. terrestris*, *Dufourea novaeangliae*, *Eufriesea mexicana*, *Habropoda laboriosa*, *Lasioglossum albipes*, *Megachile rotundata*, and *Melipona quadrifasciata*), for 6,369 of the 7,077 genes contained in the *rsd* file (evolutionary rates of differentially expressed genes available in Additional file Tables). Relative evolutionary rates are quantified as the average of inter-species protein sequence identities normalized to the average identity of all inter-species orthologs. We compared the rates of genes with no significant changes in expression (*N* = 6,069) to the rates of genes in the three categories: differentially-regulated, up-regulated and down-regulated (*N* = 155, 49 and 96, respectively), using a Kruskal-Wallis tests and a Dunn’s test with Benjamini-Hochberg corrected p-values for multiple pairwise comparisons.

### Specific response to pathogens

To detect the specific response of honey bees to pathogens, we developed a novel method which applies the same rank product analysis as above, but on ranks that reflect the degree of similarity between a gene’s expression profile and a custom expression profile across the 19 datasets. In other words, we identified genes whose expression profile is most similar to a theoretical expression profile corresponding to selected parameters. We called this method *DiRank*, for directed rank product analysis (R code available at https://idata.idiv.de/DDM/Data/ShowData/35). For the current study, we identified genes that were specifically expressed in (i) abdominal tissues (i.e. gut, fat body or whole abdomens) after *Nosema* spp. infections (datasets #3-9, see Table 2) or (ii) in all tissues after RNA virus infection or *Varroa* parasitism, as mites transmit RNA viruses (datasets #12-19). We thereby designed theoretical gene expression profiles across the 19 datasets. As an example, to identify genes specifically regulated in abdominal tissues after *Nosema* infection, we designed two custom profiles, one profile of increased expression in abdominal tissues of *Nosema* infected bees, with no changes of expression levels in other datasets, and a second profile of decreased expression in abdominal tissues of *Nosema* infected bees, with no changes of expression levels in other datasets. In the ranked *rsd* file, differential expression thresholds was defined, such as genes with only slight expression changes, with relative ranks ranging from 0.3 to 0.7, were considered as non-differentially expressed and assigned a relative rank of 0.5. These thresholds reduced the impact of small variation in expression levels on the analysis.

**Fig. 5:**
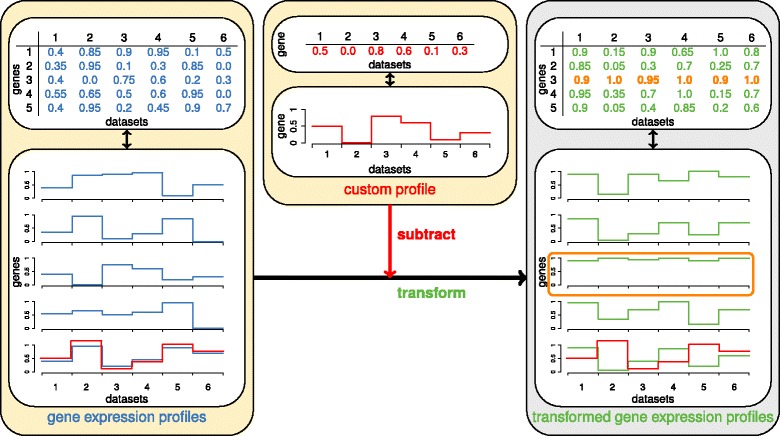
Methodological workflow of the directed rank product analysis (*DiRank*). This new method aims to identify genes with similar expression profile to a theoretical or observed profile of another gene. Gene expression values and profiles (*gep*s) (shown in *blue*) and custom profile (*cp*) (shown in *red*), consisting of relative rank values, serve as input (*yellow* boxes). In rectangular matrices, gene expression values are reported in rows, while columns represent the transcriptome datasets. A custom profile can either be a user-defined profile or an existing gene expression profile. The directed rank product analysis aims to identify genes with a similar expression profile to the custom profile and to assign associated p-values. The custom profile is subtracted from each of the gene expression profiles and each difference (*gep* - *cp*) is transformed by 1 -| *gep* - *cp*|. Transformed gene expression values and corresponding profiles are shown in green in the grey box. These transformed gene expression values are then used as input data for a rank product analysis. As an example, the transformed gene expression values surrounded by an orange frame are ranked on top by the rank product analysis as the original gene expression profile was the most similar (before transformation) to the custom profile

We then subtracted the values of the custom profile from each of the 7,077 gene expression profiles, generating a differential profile for each gene. Genes with expression profiles similar to the custom profile received a majority of differential expression values of 0, while very dissimilar profiles tended towards values of 1 or −1. Absolute values were then subtracted from 1, and genes showing a similar profile to the custom profile tended to have values close to 1 and dissimilar profiles close to 0. Using the rank product approach, as thus described, we identified genes that significantly followed the custom profile. Figure 5 illustrates the method of the directed rank product applied here. In addition, the same analyses adapted for identification of the most dissimilar (i.e. opposite) profiles were performed (Additional file 1: Figure S9) to build a gene co-expression network (see below).

All directed rank product analyses resulted in a list of genes annotated with their rank product values, their ranks according to the rank product value, *p*-value, corrected *p*-value, and ranks across the 19 transcriptome datasets. We used a cut-off uncorrected *p*-value < 0.01 to identify genes specifically regulated under the selected parameters (*Nosema* in abdominal tissues or *Varroa*/virus) and to identify the associated overrepresented GO terms.

### Gene co-expression networks

Following an iterative process, we defined the expression profile of each gene as a custom profile for our *DiRank* method, so as to retrieve genes with similar expression across datasets, and the inverse of each gene profile (i.e. inverted) to identify genes with an opposite expression profile (see Additional file 1: Figure S9).

For each of the 7,077 genes from the ranked *rsd* file we obtained a list of other genes that showed a similar or an opposite expression profile. We then reconstructed the gene co-expression network using inter-gene connection falling under a Benjamini-Hochberg corrected p-value cut-off of 0.05, after permutation test (*N* = 1,000). We visualized the network using Cytoscape [73], with genes as the ‘nodes’ of the network and gene interactions as the ‘edges’ between nodes, while a ‘module’ is a subset of interconnect nodes. We defined highly connected genes as the top 5% most connected nodes, which we termed ‘hub genes’.

To identify novel candidate genes involved in immune regulation, we collected all interactions involving canonical immune genes based on the literature [35] (listed in Additional file 2: Table ST14).

## Additional files

Additional file 1: Figure S1-S9. This file includes supplementary figures documenting our multidimensional scaling analysis results, a heat map of the differential expression of the 7,077 genes across the 19 datasets, a Venn diagram of differentially expressed genes, the expression profile of the gene coding for *hymenoptaecin*, the distribution of genes according to their number of inter-gene connections, the degree of connectivity of differentially expressed genes, the process of gene selection for this study, the distribution of genes’ differential expression across datasets, and a diagram illustrating our new bioinformatics approach. (PDF 683 kb)
Additional file 2: Table ST1. List of 7,077 genes ordered by their rank product after the rank product analysis looking for up-regulated genes. Genes ordered from higher ranks (up-regulated) to lower ranks (non-regulated). **Table ST2.** List of 7,077 genes ordered by their rank product after the rank product analysis looking for down-regulated genes. Genes ordered from higher ranks (down-regulated) to lower ranks (non-regulated). **Table ST3.** List of 7,077 genes ordered by their rank product after the rank product analysis looking for differentially-regulated genes. Genes ordered from higher ranks (differentially-regulated) to lower ranks (non-regulated). **Table ST4.** List of 7,077 genes ordered by their rank product after the directed rank product analysis looking for up-regulated genes in abdominal tissue, after *Nosema* infection. **Table ST5.** List of 7,077 genes ordered by their rank product after the directed rank product analysis looking for down-regulated genes in abdominal tissue, after *Nosema* infection. **Table ST6.** Functional analysis (GO slim) based on top up-regulated genes in abdominal tissues (gut, fat body or all abdomen) upon infection by *Nosema*. Cut-off < 0.01 uncorrected p-value, genes from S4 Table. **Table ST7.** Functional analysis (GO slim) based on top down-regulated genes in abdominal tissues (gut, fat body or all abdomen) upon infection by *Nosema.* Cut-off < 0.01 uncorrected p-value, genes from S5 Table. **Table ST8.** List of 7,077 genes ordered by their rank product after the directed rank product analysis looking for up-regulated genes after RNA virus infection and *Varroa* infestation. **Table ST9.** List of 7,077 genes ordered by their rank product after the directed rank product analysis looking for down-regulated genes after RNA virus infection and *Varroa* infestation. **Table ST10.** Functional analysis (GO slim) based on top up-regulated genes upon infection by RNA virus and *Varroa* infestation. Cut-off < 0.01 uncorrected p-value, genes from S8 Table. **Table ST11.** Functional analysis (GO slim) based on top down-regulated genes upon infection by RNA virus and *Varroa* infestation. Cut-off < 0.01 uncorrected p-value, genes from S9 Table. **Table ST12.** List of the 209 highly connected (hub) genes with at least 34 inter-gene connections. **Table ST13.** List of genes involved in the immune gene network (Fig. 4C). **Table ST14.** List of immune genes used to construct the immune gene network (Fig. 4C). **Table ST15.** Experimental procedure and description of datasets. (XLSX 9947 kb)

## Abbreviations

Ago: Argonaute
AMP: Antimicrobial peptide
Apd: Apidermin
B-gluc1: Beta-1,3-glucan recognition protein 1
BQCV: Black queen cell virus
Def: Defensin
Drk: Downstream of receptor kinase
DWV: Deformed wing virus
EF1-alpha: Elongation factor 1-alpha
fsd: Full synthesised dataset
GMC: Glucose-methanol-choline
GO: Gene ontology
iap2: Inhibitor of apoptosis 2
IAPV: Israeli acute paralysis virus
IMD: Immunodeficiency
IRP30: Immune-responsive protein 30
JAK/STAT: Janus kinase/signal transducer and activator of transcription
Lys: Lysozyme
MAPK: Mitogen-activated protein kinase
Mrjp: Major royal jelly proteins
Mvl: Malvolio
NADH: Nicotinamide adenine dinucleotide H
NF-kB: Nuclear factor kappa B
p.i.: Post infection
PGRP: Peptidoglycan recognition proteins
rel: Relish
RNA: Ribonucleic acid
RNA-seq: Ribonucleic acid sequencing
rsd: Restricted synthesised dataset
Serpin: Serine proteinase inhibitors
Socs: Suppressor of cytokine signalling
SP: Serine protease
Vg: Vitellogenin
