# Erratum to: Unity in defence: honeybee workers exhibit conserved molecular responses to diverse pathogens

**DOI:** 10.1186/s12864-017-3624-7

**Published:** 2017-03-23

**Authors:** Vincent Doublet, Yvonne Poeschl, Andreas Gogol-Döring, Cédric Alaux, Desiderato Annoscia, Christian Aurori, Seth M. Barribeau, Oscar C. Bedoya-Reina, Mark J. F. Brown, James C. Bull, Michelle L. Flenniken, David A. Galbraith, Elke Genersch, Sebastian Gisder, Ivo Grosse, Holly L. Holt, Dan Hultmark, H. Michael G. Lattorff, Yves Le Conte, Fabio Manfredini, Dino P. McMahon, Robin F. A. Moritz, Francesco Nazzi, Elina L. Niño, Katja Nowick, Ronald P. van Rij, Robert J. Paxton, Christina M. Grozinger

**Affiliations:** 1 German Centre for Integrative Biodiversity Research (iDiv) Halle-Jena-Leipzig, Leipzig, Germany; 2 Centre for Ecology and Conservation, University of Exeter, Penryn, UK; 3 Institute of Computer Science, Martin Luther University Halle-Wittenberg, Halle (Saale), Germany; 4 Technische Hochschule Mittelhessen, Gießen, Germany; 5 INRA, UR 406 Abeilles et Environnement, Avignon, France; 6 Dipartimento di Scienze AgroAlimentari, Ambientali e Animali, Università degli Studi di Udine, Udine, Italy; 7 Institute of Life Sciences, University of Agricultural Sciences and Veterinary Medicine, Cluj-Napoca, Romania; 8 Department of Biology, East Carolina University, Greenville, NC USA; 9 Center for Comparative Genomics and Bioinformatics, Pennsylvania State University, State College, PA USA; 10 School of Biological Sciences, Royal Holloway University of London, Egham, Surrey UK; 11 Department of Biosciences, Swansea University, Swansea, UK; 12 Department of Plant Sciences and Plant Pathology, Montana State University, Bozeman, MT USA; 13 Department of Entomology, Center for Pollinator Research, Pennsylvania State University, State College, PA USA; 14 Department of Molecular Microbiology and Bee Diseases, Institute for Bee Research, Hohen Neuendorf, Germany; 15 Department of Microbiology and Epizootics, Freie Universität Berlin, Berlin, Germany; 16 Department of Fisheries, Wildlife, and Conservation Biology, The Monarch Joint Venture, University of Minnesota, St. Paul, MN USA; 17 Department of Molecular Biology, Umeå University, Umeå, Sweden; 18 Institute for Biology, Martin Luther University Halle-Wittenberg, Halle (Saale), Germany; 19 School of Biological Sciences, Queen’s University Belfast, Belfast, UK; 20 Institute of Biology, Freie Universität Berlin, Berlin, Germany; 21 Department for Materials and Environment, BAM Federal Institute for Materials Research and Testing, Berlin, Germany; 22 Department of Entomology and Nematology, University of California, Davis, CA USA; 23 Department of Computer Science, TFome Research Group, Bioinformatics Group, Interdisciplinary Center of Bioinformatics, University of Leipzig, Leipzig, Germany; 24 Paul-Flechsig-Institute for Brain Research, University of Leipzig, Leipzig, Germany; 25 Department of Medical Microbiology, Radboud Institute for Molecular Life Sciences, Radboud University Medical Center, Nijmegen, The Netherlands; 26 Present address: MRC IGMM, University of Edinburgh, Western General Hospital, Edinburgh, UK; 27 Present address: MRC Functional Genomics Unit, Department of Physiology, Anatomy and Genetics, University of Oxford, South Parks Road, Oxford, UK; 28 Present address: International Centre of Insect Physiology and Ecology (icipe), Environmental Health Theme, Nairobi, Kenya

## Erratum

After the publication of this work [1] it was noticed that there was a typesetting error in figure 5 where two additional red lines were added in the sections “gene expression profiles” and “transformed gene expression profiles”. The original article was corrected to remove these lines.
