# Supplementary material for: Unity in defence: honeybee workers exhibit conserved molecular responses to diverse pathogens
Source: BMC Genomics. 2017 Mar 2;18:207. doi: 10.1186/s12864-017-3597-6 (PMC5333379; doi:10.1186/s12864-017-3597-6)

Supplementary Figures S1-S9

Unity in defence: honeybee workers exhibit conserved molecular responses to diverse pathogens

Vincent Doublet, Yvonne Poeschl, Andreas Gogol-Döring, Cédric Alaux, Desiderato Annoscia, Christian Aurori, Seth M. Barribeau, Oscar Bedoya-Reina, Mark J. F. Brown, James C. Bull, Michelle L. Flenniken, David A. Galbraith, Elke Genersch, Sebastian Gisder, Ivo Grosse, Holly L. Holt, Dan Hultmark, H. Michael G. Lattorff, Yves Le Conte, Fabio Manfredini, Dino P. McMahon, Robin F. A. Moritz, Francesco Nazzi, Elina L. Niño, Katja Nowick, Ronald P. van Rij, Robert J. Paxton and Christina M. Grozinger

Supplementary Figure S1: Multidimensional scaling analysis. Each dot represents one of the 19 genome-wide differential gene expression data used in this synthesis. Numbers refer to the nomenclature in Table 2 and colours illustrate the different studies that generated the data. The clustering of transcriptome datasets from the same studies (i.e. colours) shows the high impact of study design on the data.

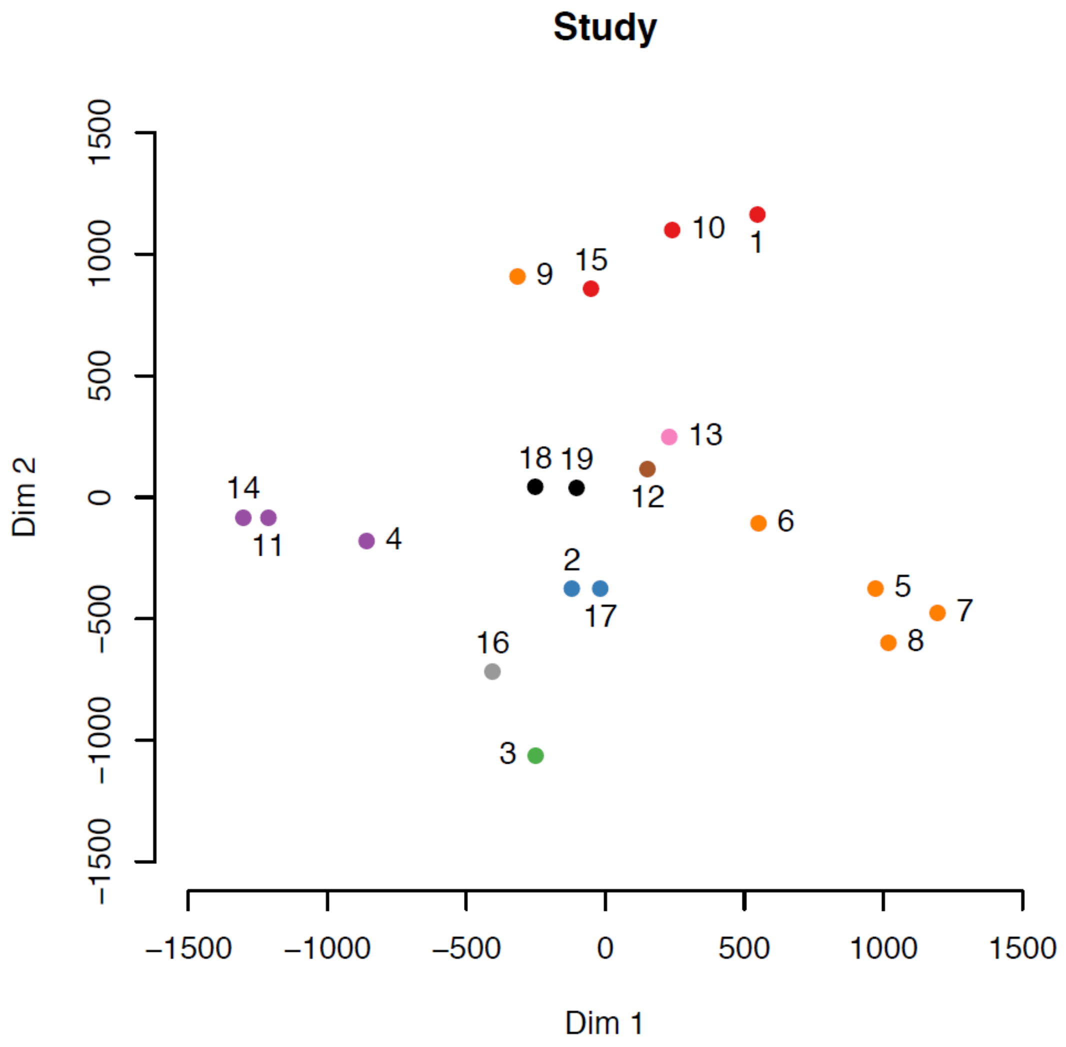

Supplementary Figure S2: Heat map displaying the computed rank product values for all 7,077 genes across the 19 transcriptome datasets. Genes are ordered vertically from most up-regulated (in orange) to most down-regulated (blue), and horizontally (x-axis) according to Table 2 (N= *Nosema*; N/V= mix-infection *Nosema* + RNA Virus; V= RNA viruses; M= *Varroa* mite). Each row represents the differential expression of the same gene across all 19 datasets. Genes mean rank values are displayed in the right column of the heat map.

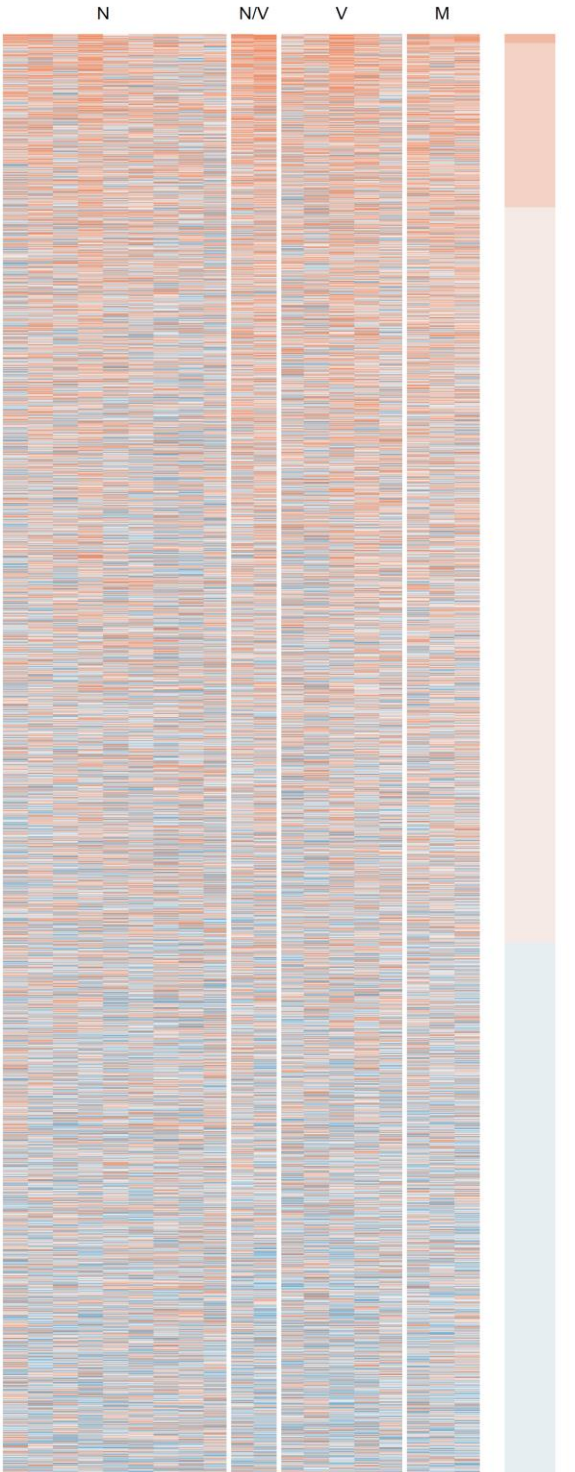

N
N/V
V
M
1 2 3 4 5 6 7 8 9 10 11 12 13 14 15 16 17 18 19 mean rank

Supplementary Figure S3: Venn diagram showing the number of genes exhibiting significant differential expression across the 19 datasets. Circles include the three categories: up-regulated (purple), down-regulated (green) and differentially-regulated (blue), with number of genes in all categories. Total number of significantly differentially expressed genes = 344.

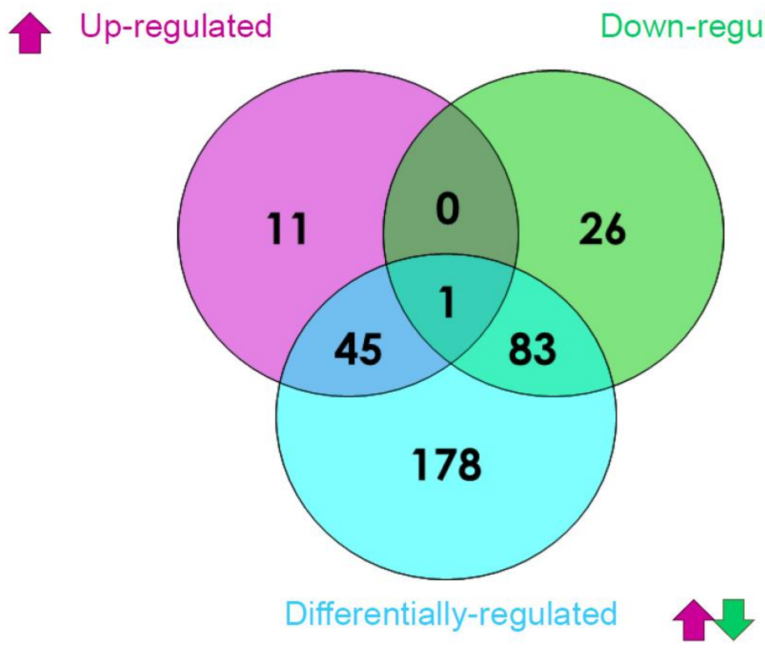

Supplementary Figure S4: Differential expression profile of the gene *hymenoptaecin* (*LOC406142*) across the 19 transcriptome datasets (in red). The extreme up- and down-regulation of this gene justifies its presence in all categories of our rank product analysis, as a significantly up-regulated gene, significantly down-regulated gene, and logically, therefore, as a differentially regulated gene. The blue shade shows the inter-quartile range of differential expression of significantly differentially regulated genes across all datasets, while the blue line represents the median values.

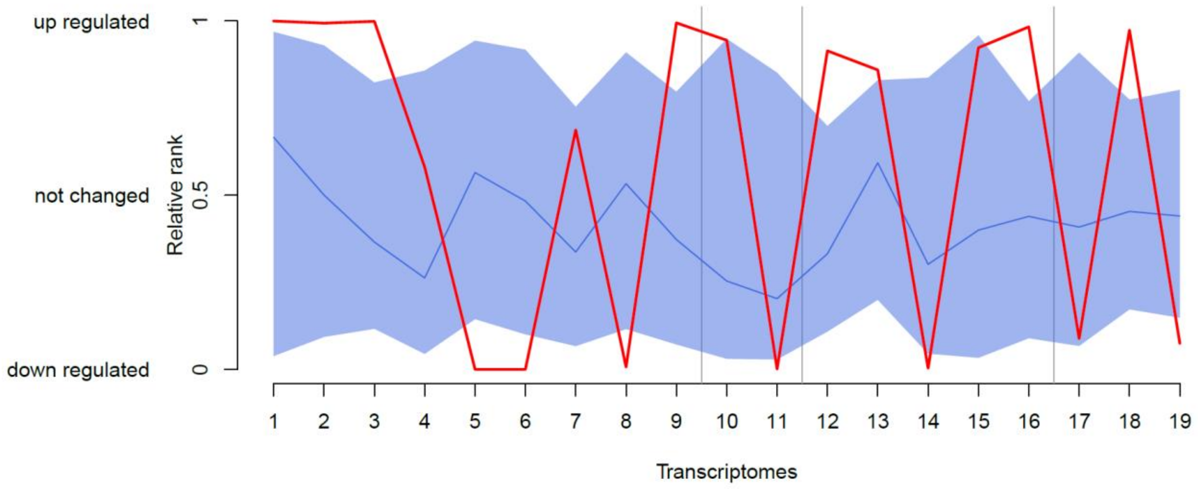

Supplementary Figure S5: Genes sorted by their number of connections in decreasing order. Black circles show non-differently expressed genes (N = 6,733), and red crosses show significantly differentially expressed genes (N = 344). The vertical blue line shows the top 5% most connected genes (N = 209), which have at least 34 connections to other genes (highly similar or highly dissimilar expression profiles).

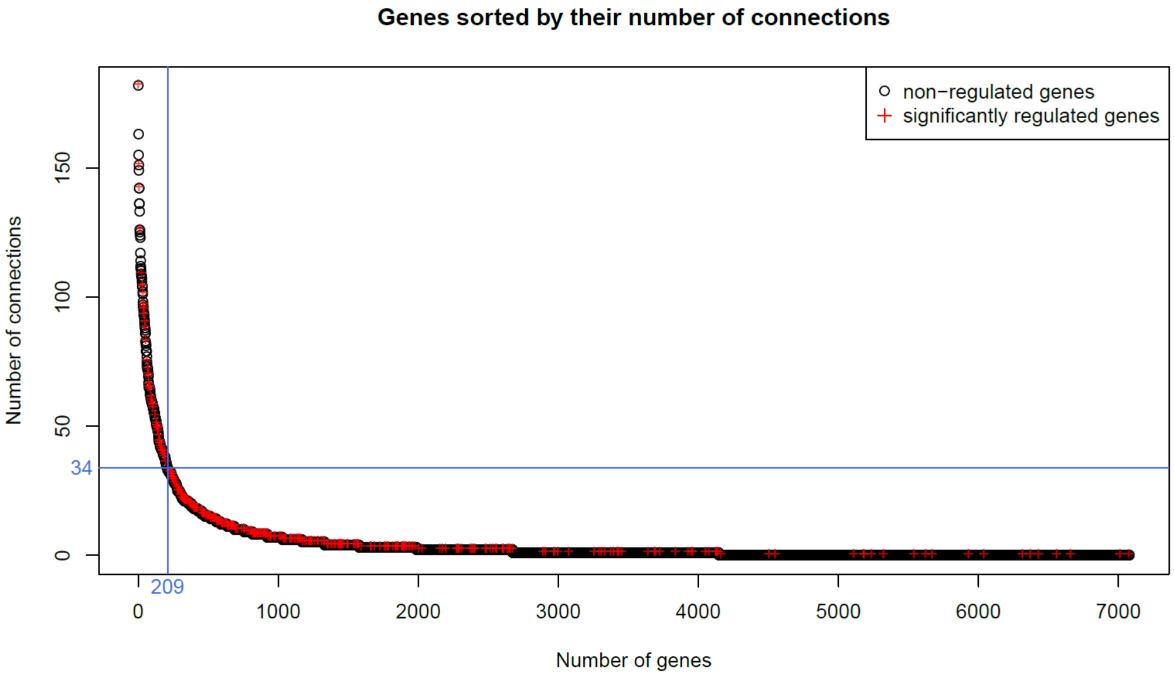

Supplementary Figure S6: Degree of connectivity of genes. Number of connections is log_2 transformed, and represented for the four categories of genes of the rank product analysis: non-differentially expressed genes (grey), differentially-regulated, up and down (green), up-regulated (orange) and down-regulated genes (blue). All categories of significantly regulated genes are more connected that non-significantly differentially expressed genes.

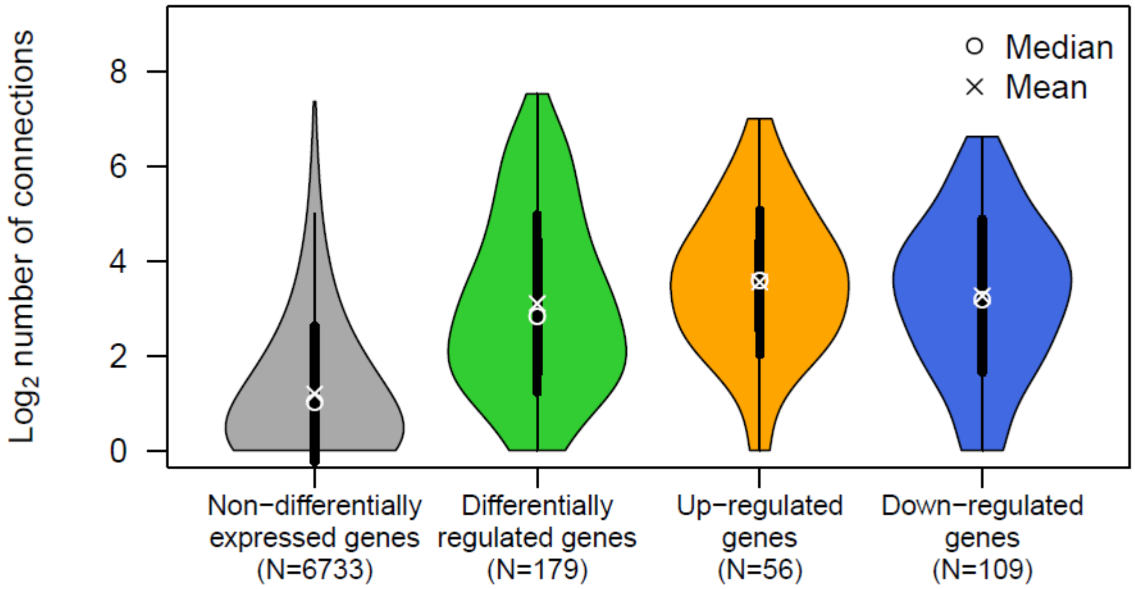

Supplementary Figure S7: Gene selection for analysis. Distribution of the 11,165 annotated honey bee genes with zero to 19 missing values across the 19 transcriptome datasets, with above number of genes for each category (top). Cumulative distribution of genes in the same categories, illustrating the addition of 2,159 genes with one, two or three missing values (in grey) to increase our sample size to 7,077 genes, and reach 63% (above number and percentage) of the annotated genes of the honey bee genome for our analyses (bottom).

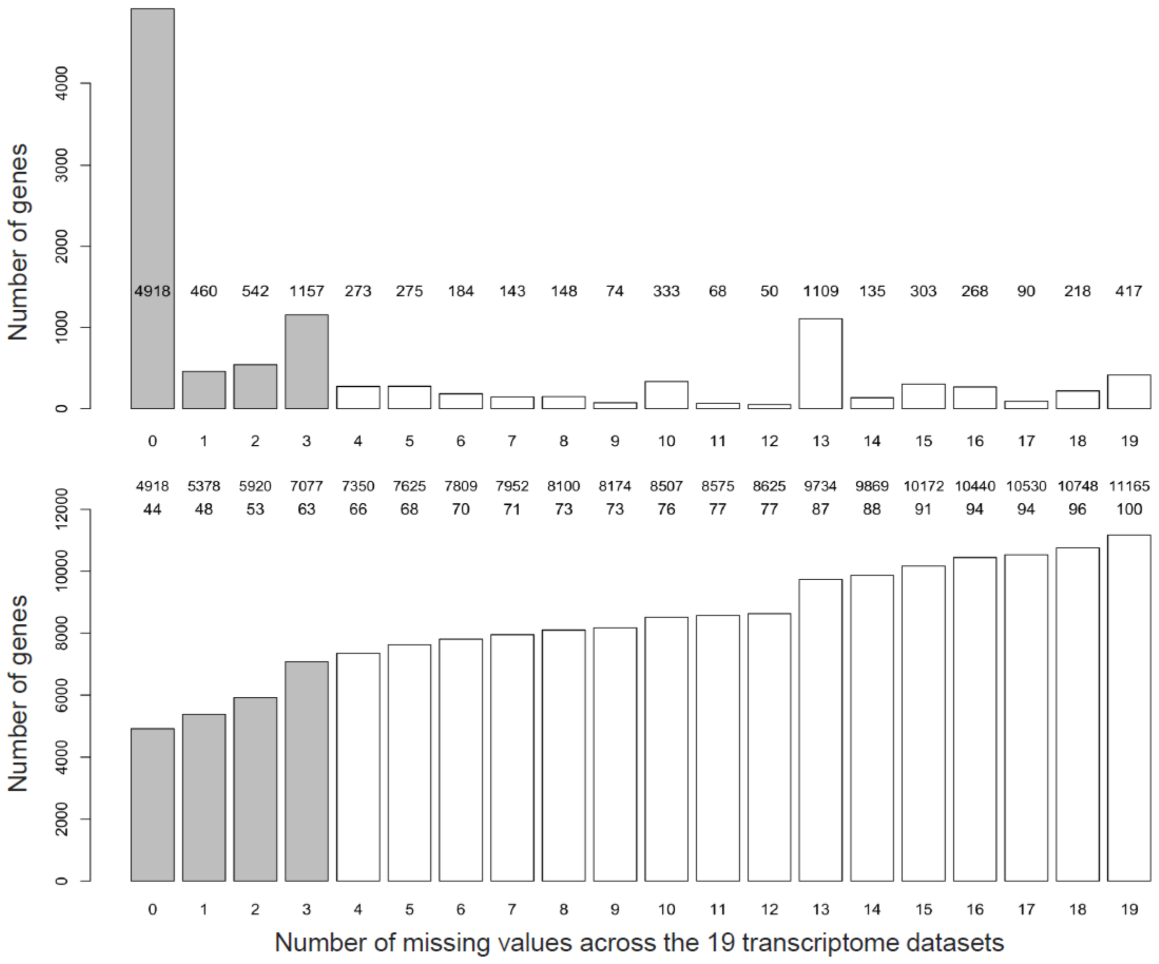

Supplementary Figure S8: Boxplot showing the gene expression level (in log_2 fold changes between control and infected individuals) across the 19 transcriptome datasets, ordered according to pathogen/parasite type. Differential gene expression values acquired by microarray (grey background) display lower amplitude than whole transcriptome sequencing (white background) due to the difference in sensitivity of the assays, thus justifying the use of the rank product approach. A shift of the median value of honey bee gene differential expression from 0 in dataset #16 is due to the high number of virus reads in the transcriptome dataset that influenced data normalization. The use of rank product analysis corrects for such discrepancies resulting from normalization across studies. N=*Nosema*; N/V= *Nosema*/Virus co-infection; V= RNA viruses; M= *Varroa* mite. Numbers in x-axis refer to the nomenclature of the transcriptome datasets in Table 2.

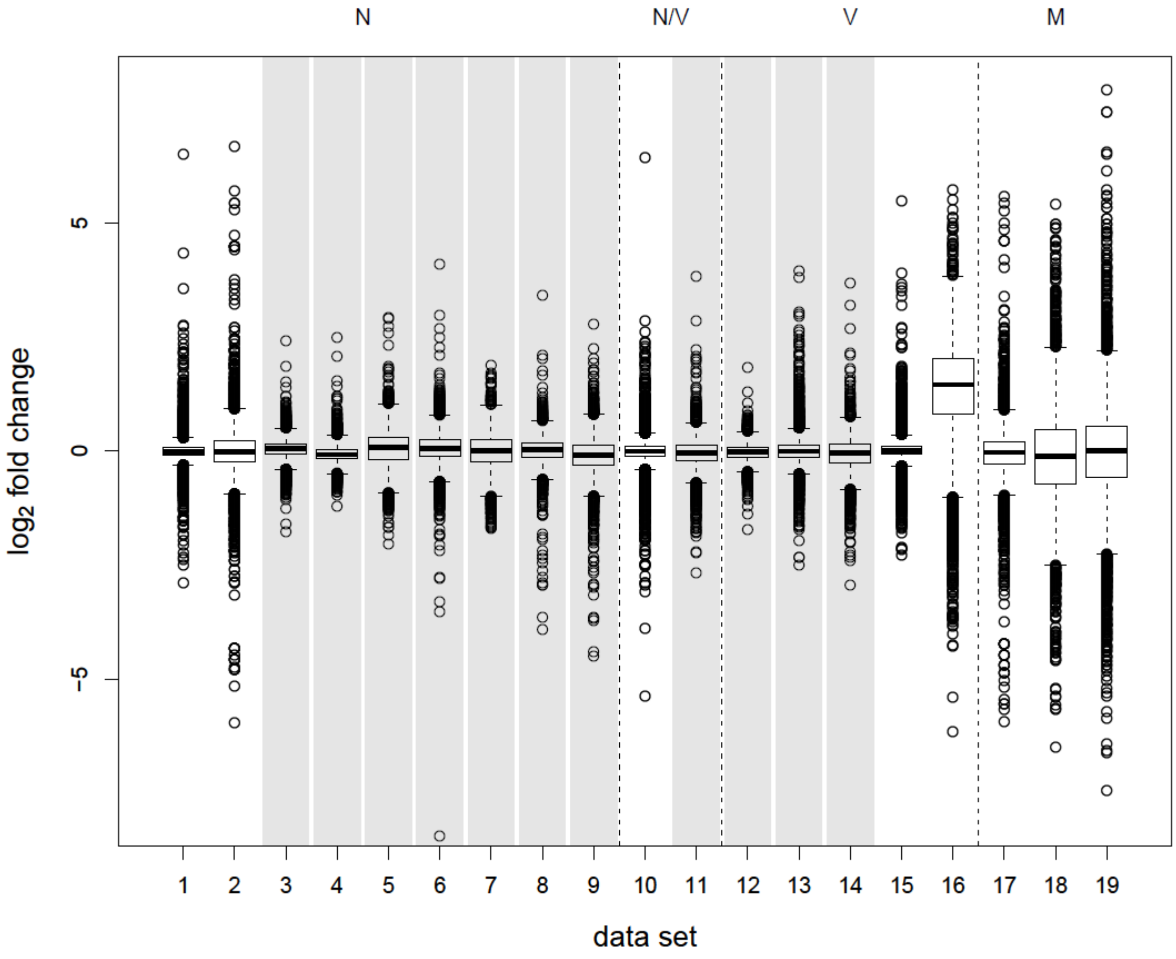

Supplementary Figure S9: Diagram illustrating the directed rank product analysis adapted for identification of opposite gene expression profiles. (A) The first step to identify opposite profiles from a focal gene is the transformation of the focal gene expression profile by inversion. This inverted gene expression profile will become the custom profile for the directed rank product. **(B)** After inversion of the focal gene expression profile, the directed rank product analysis is identical to the description in Fig 1. Gene expression values and profiles (*gep*s) (shown in blue) and the inverted profile (*ip*) (shown in red) consisting of relative rank values served as input (yellow boxes). The directed rank product analysis aims at identifying genes that show a similar profile as the custom profile and assigning associate p-values. The custom profile is subtracted from each of the gene expression profiles and each difference (*gep* - *ip*) is transformed by 1 -| *gep* - *ip*|. The transformed gene expression values and corresponding profiles are shown in green colour in the grey box. The transformed gene expression values are further used as input data for a rank product analyses. The transformed gene expression profile surrounded by an orange frame shows the most similar profile to the inverted profile (i.e. the most dissimilar profile to the focal gene expression profile) and will be ranked first after rank product analysis.

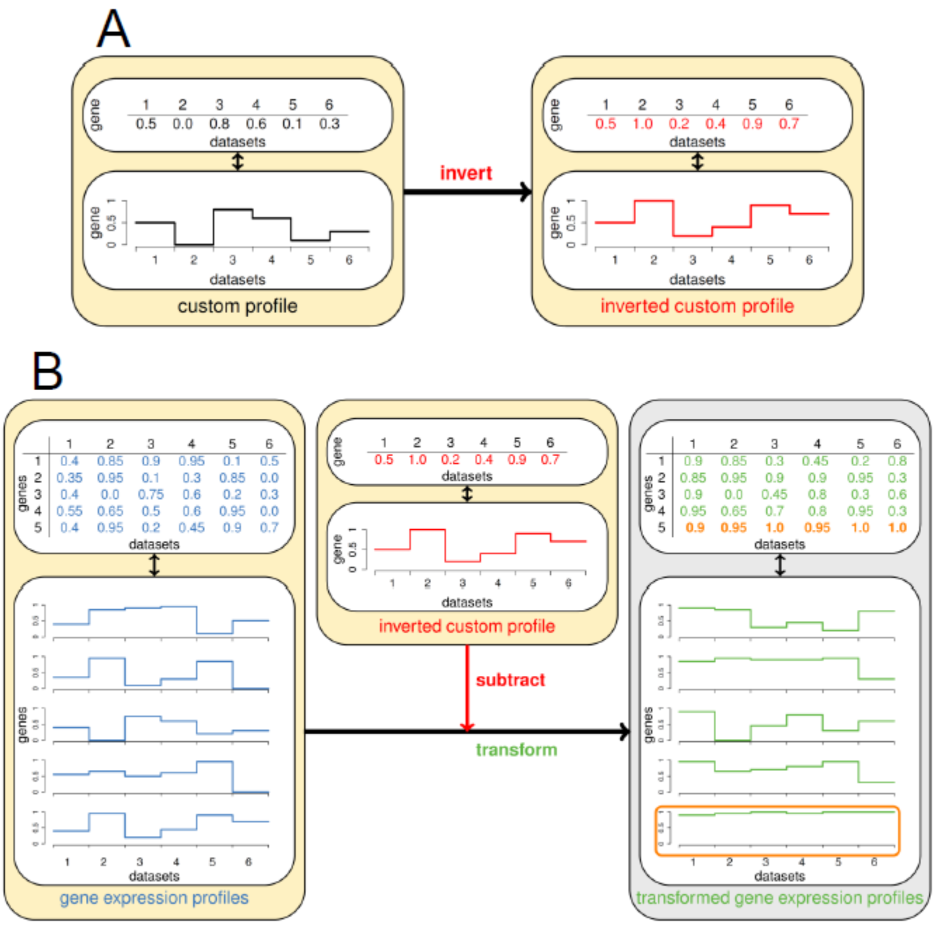

Supplement: Additional file 1: Figure S1-S9. — This file includes supplementary figures documenting our multidimensional scaling analysis results, a heat map of the differential expression of the 7,077 genes across the 19 datasets, a Venn diagram of differentially expressed genes, the expression profile of the gene coding for hymenoptaecin, the distribution of genes according to their number of inter-gene connections, the degree of connectivity of differentially expressed genes, the process of gene selection for this study, the distribution of genes’ differential expression across datasets, and a diagram illustrating our new bioinformatics approach. (PDF 683 kb) [file 12864_2017_3597_MOESM1_ESM.pdf]
